# Neuraxial Anesthesia for an Open Low Anterior Rectal Resection: Tip the Scales in Patient’s Favor

**DOI:** 10.7759/cureus.57094

**Published:** 2024-03-27

**Authors:** Francesco Marrone, Pierfrancesco Fusco, Luca Lepre, Michela Giulii Capponi, Alessandra Villani, Saverio Paventi, Marco Tomei, Roberto Starnari, Carmine Pullano

**Affiliations:** 1 Department of Anesthesiology and Critical Care, Santo Spirito Hospital, Rome, ITA; 2 Department of Anesthesia, Santo Spirito Hospital, Rome, ITA; 3 Department of Anesthesia and Intensive Care Unit, San Filippo e Nicola Hospital, Avezzano, ITA; 4 Department of Surgery, Santo Spirito Hospital, Rome, ITA; 5 Department of Anesthesiology, Santo Spirito Hospital, Rome, ITA; 6 Department of Anesthesiology and Critical Care, Azienda Sanitaria Locale Roma 1, Rome, ITA; 7 Department of Anesthesiology, Istituto Nazionale di Ricovero e Cura per Anziani (INRCA), Ancona, ITA; 8 Department of Anesthesia, Villa Pia Clinic, Rome, ITA

**Keywords:** post-operative pain management, colo-rectal cancer, hypobaric spinal technique, combined spinal epidural, neuraxial anaesthesia

## Abstract

We present the case of a successful application of combined spinal-epidural anesthesia for a geriatric patient undergoing open cancer surgery. The patient, affected by multiple comorbidities, was proposed for an open anterior rectal resection. The implementation of a tailored protocol, incorporating neuraxial techniques such as epidural and spinal anesthesia, facilitated optimal pain management and expedited postoperative recovery improving perioperative outcomes, and highlighting the potential benefits of such strategies in selected cases.

## Introduction

Postoperative pulmonary complications occur more frequently in patients with pre-existing pulmonary disease compared to those without such conditions. Abdominal surgeries carry one of the highest rates of surgical pulmonary complications. Consequently, many patients with severe pulmonary impairment may be not eligible for elective abdominal surgeries due to the risk of worsening their pulmonary function. These complications may depend on the impact of general anesthesia (GA) and mechanical ventilation on compromised lungs, compounded by the challenges in managing postoperative pain, which exacerbates pulmonary function impairment due to increased analgesic (opioids) requirements. Regional and neuraxial anesthesia (NA) may be an attractive alternative for major abdominal procedures in high-risk and frail patients with cardiovascular and respiratory issues, especially when GA poses an increased risk of adverse outcomes [1,2]. In this case, we provided the description of using a combined spinal-epidural anesthesia in a geriatric patient with many comorbidities who underwent open rectal surgery for cancer showing that surgery may be safely and effectively performed with a smooth postoperative recovery and without complications also in very sick patient.

## Case presentation

**Figure 1:**
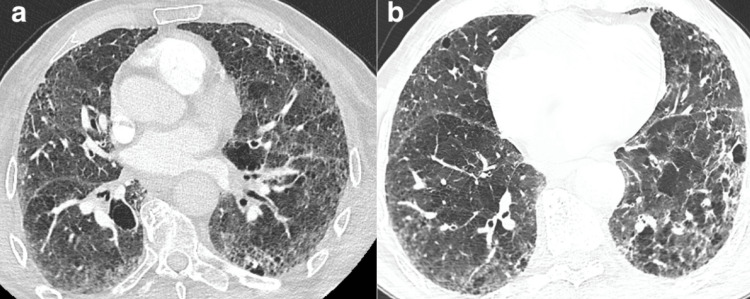
Computed tomography (CT) scan of lungs (a, b) Two-level thoracic CT scans showing lung emphysema.

Herein, we present the case of an 82-year-old male patient classified as ASA-III (American Society of Anesthesiologists Physical Status Classification) with a weight of 81 kg and height of 170 cm (BMI 28 kg.m^-^²). The patient underwent combined spinal-epidural (CSE) anesthesia as an alternative method for an open low anterior resection of rectal cancer. The patient, a smoker, had a medical history that included abdominal aorta ectasia, hypertension, atrial fibrillation treated with edoxaban, and chronic obstructive airway disease (emphysema), resulting in hypoxemic failure (Figure 1). He had a history of dyspnea on minimal exertion (New York Heart Association (NYHA) class III). Due to the concerns that GA might contribute to further respiratory failure, necessitating prolonged ventilation or intensive care management, we opted for NA after discussing issues with the patient, with GA held as a rescue strategy. The patient provided written informed consent, and the surgical team was aware of the chosen approach. An arterial cannula was inserted into a radial artery for blood pressure and gas monitoring. Colloids (gelatin) (500 mL) were administered before the procedure. The patient received oxygen via nasal cannula (4 L.min^-1^). Vital parameters were monitored during the procedure. First, an epidural catheter (T12-L1) was aseptically placed using an 18G Tuohy-needle and median approach, and a testing dose was given (2% lidocaine 3 mL). Subsequently, an L1-L2 spinal puncture (25G Whitacre needle, B. Braun, Melsungen, Germany) was performed via a median approach, providing 0.5% hyperbaric bupivacaine 1.5 mL (7.5 mg). After that, by rotating the bevel of the needle, 0.25% hypobaric levobupivacaine at a dose of 10 mg (2 mL of 0.5% levobupivacaine diluted with 2 mL of sterile distilled water for a total volume of 4 mL) was administered [3]. The patient remained in a sitting position for one minute while the anesthesia level was assessed by pinprick. After that, the patient was placed in the supine position. A starting epidural bolus of 0.2% ropivacaine 5 mL was provided. Sedation with intravenous midazolam 2 mg, fentanyl 50 mcg, and dexmedetomidine (0.6 mcg.kg^-1^.h^-1^) was started. The surgical team had easy access to the surgical field (T4-S3). Prior to mobilizing the splenic flexure (one hour after incision), we administered intravenously an additional 50 mcg fentanyl and epidural 0.2% ropivacaine 10 mL. The surgery lasted 155 minutes and was uneventful. The patient received 2.5 L of intravenous crystalloids. Blood loss was approximately 150 mL. Arterial blood gas analysis didn't show respiratory acidosis, only a metabolic acidosis corrected with intravenous administration of sodium bicarbonate 50 mEq. Bradycardia or hypotension was not recorded. At the end of the surgery, the patient remained in the post-anesthesia care unit (PACU), monitored for 30 min, and then was discharged to the surgical ward with an Aldrete score of 9 (motor weakness of the lower limbs was reported) and a Ramsay sedation score of 2-3. Postoperative analgesia was managed with an epidural elastomeric pump (5 mL.h^-1^) containing 0.2% ropivacaine without opioids. Intravenous paracetamol (1 g) was administered three times daily. The patient didn’t receive blood transfusions and was discharged without complications from the hospital after 10 days.

## Discussion

Open surgery for cancer in elderly patients may result in a significant risk of morbidity and mortality and GA represents a major risk of respiratory post-operative complications. In this case, CSE anesthesia provided an alternative choice for a patient with lung failure and other comorbidities. In the literature we accessed, there was one case that drew some comparison to ours: a patient with congestive heart failure, chronic obstructive pulmonary disease (COPD), and severe scoliosis with poor lung function scheduled for an open sigmoid colectomy. A CSE was provided as an alternative anesthesia choice as the authors were worried about the risks that a general anesthetic would contribute to further respiratory failure with prolonged intensive care management and ventilation [4]. While low anterior rectal resection is commonly performed under GA with the potential role of epidural analgesia intra- and postoperatively, we achieved positive results by employing CSE as an alternative technique in a very sick patient. Epidural analgesia is associated with improved health outcomes for surgical patients with COPD to reduce post-operative complications [5] and CSE anesthesia has been successfully implemented as the sole anesthetic technique in major abdominal surgeries in patients with COPD [6]. In patients with interstitial lung disease, a regional technique or NA may be considered a choice for the avoidance of GA and its complications [7]. We argue that neuraxial techniques can be safely and effectively administered with meticulous planning in frail patients with multiple comorbidities proposed for abdominal surgery when it would be preferable to avoid GA after conducting a comprehensive risk-benefit assessment. Moreover adopting this approach may not only diminish admissions to the Intensive Care Unit, thereby conserving beds for acute and trauma patients and reducing hospitalization costs during periods of resource scarcity and therapeutic constraints [8], but may also align with the Enhanced Recovery After Surgery (ERAS) concept, promoting rapid recovery and minimizing complications even in the context of open surgery. In our approach to CSE anesthesia, we did not use the "needle-through-needle" technique because failed dural puncture occasionally occurs [9] and because we consider it crucial to test peridural catheters before using them.

## Conclusions

In conclusion, CSE anesthesia revealed a safe alternative for our patient for whom GA posed major risks due to comorbidities. Managing the anesthesia in elderly patients with limited physiological reserve and accompanying comorbidities represents a challenge and necessitates a comprehensive understanding of their condition. Prior to surgery, these patients should undergo a careful assessment, and any factors that could potentially disrupt postoperative homeostasis must be addressed. In our case, CSE anesthesia offered effective surgical anesthesia while minimizing alterations to the baseline physiological state of the patient and providing substantial postoperative advantages. Further studies are needed to confirm our approach.
